# The role of bile acid metabolism in the occurrence and development of NAFLD

**DOI:** 10.3389/fmolb.2022.1089359

**Published:** 2022-12-15

**Authors:** Hao Bing, Yi-Ling Li

**Affiliations:** ^1^ Department of Gastroenterology, First Affiliated Hospital of China Medical University, Shenyang, Liaoning, China; ^2^ Department of Gastroenterology, Shengjing Hospital Affiliated with China Medical University, Shenyang, Liaoning, China

**Keywords:** non-alcoholic fatty liver disease, bile acid, metabolomics, farnesyl X receptor, gut microbiota

## Abstract

Non-alcoholic fatty liver disease (NAFLD) has become one of the important causes of cirrhosis and liver cancer, resulting in a huge medical burden worldwide. Currently, effective non-invasive diagnostic indicators and drugs for NAFLD are still lacking. With the development of metabolomics technology, the changes in metabolites during the development of NAFLD have been gradually revealed. Bile acid (BA) is the main endpoint of cholesterol metabolism in the body. In addition, it also acts as a signaling factor to regulate metabolism and inflammation in the body through the farnesyl X receptor and G protein-coupled BA receptor. Studies have shown that BA metabolism is associated with the development of NAFLD, but a large number of animal and clinical studies are still needed. BA homeostasis is maintained through multiple negative feedback loops and the enterohepatic circulation of BA. Recently, treatment of NAFLD by interfering with BA synthesis and metabolism has become a new research direction. Here, we review the changes in BA metabolism and its regulatory mechanisms during the development of NAFLD and describe the potential of studies exploring novel non-invasive diagnostic indicators and therapeutic targets for NAFLD based on BA metabolism.

## 1 Introduction

Non-alcoholic fatty liver disease (NAFLD) is the most common chronic liver disease (Powell et al., 2021), and the disease spectrum ranges from steatosis to non-alcoholic steatohepatitis (NASH) to liver cirrhosis (LC) or directly to hepatocellular carcinoma (HCC) (Huang et al., 2021). NAFLD is the fastest growing cause of HCC worldwide, and NASH will inevitably become the most common cause of HCC in many countries in the near future (Ioannou, 2021). In addition to the risk of adverse events in the liver such as LC and HCC, NAFLD significantly increased the occurrence of cardiovascular events and non-liver adverse events, such as malignancies at other sites and kidney disease (Friedman et al., 2018; Kasper et al., 2021).

The gold standard for NAFLD diagnosis is pathological biopsy, but this examination is invasive. The identification of reliable, non-invasive and accurate diagnostic indicators is very urgently needed. Metabolomics provides new ideas for the study of NAFLD pathophysiological mechanisms, the development of accurate diagnostic methods, and the identification of therapeutic targets. Metabolomics mainly detects small metabolites in the body (molecular weight <2000 Da) (Bauermeister et al., 2022). Changes in lipid products, amino acids, bile acids (BAs), glutathione and other related metabolites occur during the development of NAFLD (Kalhan et al., 2011). The role of BA metabolism in NAFLD has attracted attention. In recent years, continuous research on metabolomics in NAFLD has provided evidence for metabolic alterations during the development of NAFLD and has the potential to reveal novel non-invasive biomarkers (Di Mauro et al., 2021) and intervention therapies (Perakakis et al., 2020).

## 2 BA metabolism and regulation in humans and mice

**FIGURE 1:**
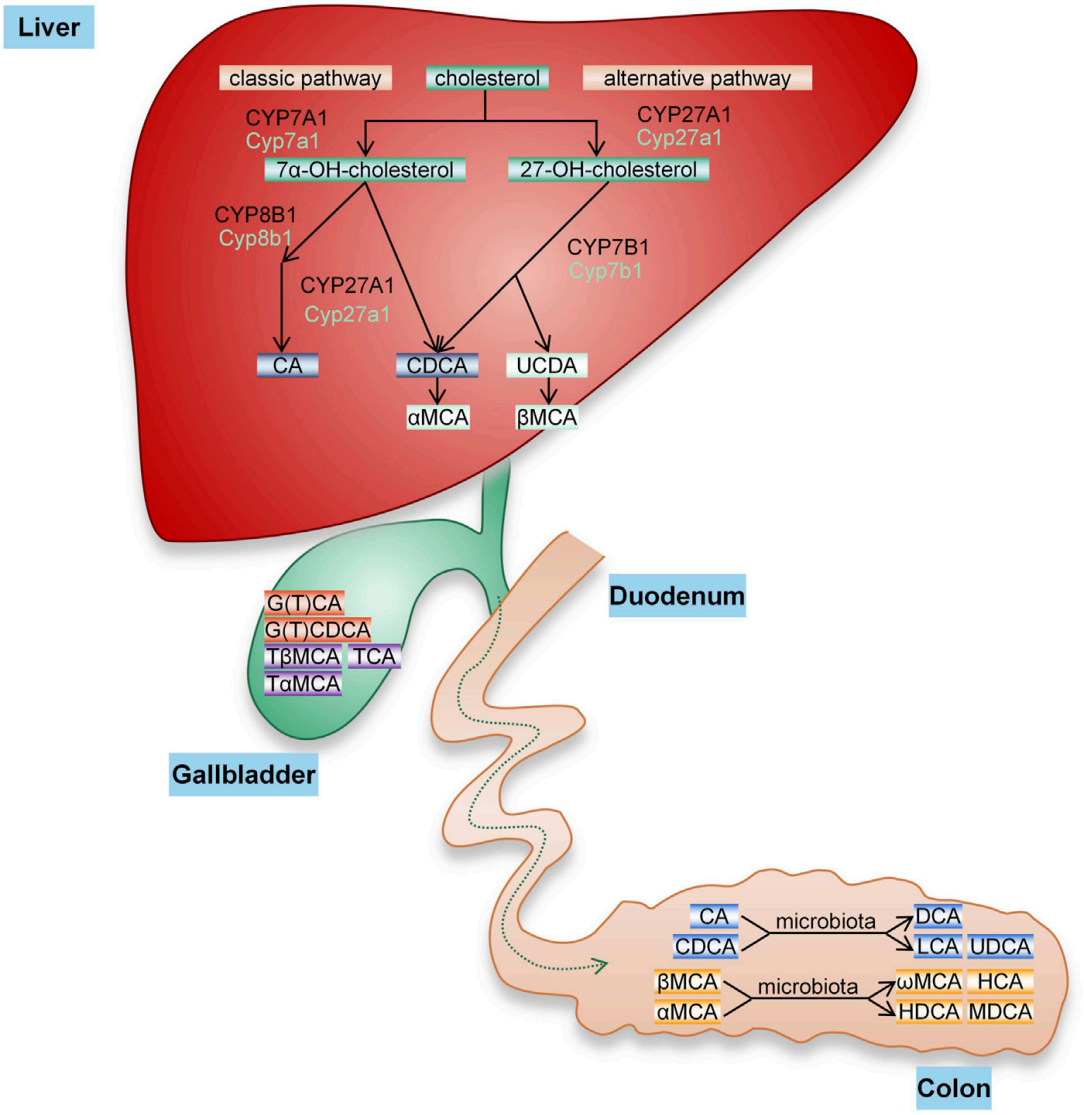
BA synthesis. The conversion of cholesterol into BA in the liver occurs through two pathways. The classical pathway of BA synthesis is initiated by CYP7A1, while the alternative pathway is initiated by CYP27A1. CA and CDCA are two main primary BAs synthesized in the human liver that are secreted into bile, bound to glycine or taurine and finally excreted into the intestine. In the gut, these primary BAs are subjected to bacterial bile salt hydrolases and dehydroxylases to produce secondary BAs, including DCA, LCA, and UDCA (rarely). In mice, CA is produced by the classical pathway, and CDCA and UDCA generated by the alternative pathway are converted to α-MCA and β-MCA and then form ω-muricholic acid (ω-MCA), hyocholic acid (HCA), dehydrocholic acid (DHCA) and murideoxycholic acid (MDCA) through the action of the intestinal flora.

BA is the general name of cholanic acid, an amphipathic steroid molecule derived from cholesterol catabolism in hepatocyte cells, and it is divided into conjugated bile acid (CBA) and free bile acid (FBA). BA synthesis involves two pathways (Figure 1): the classical pathway activated by cholesterol 7a-hydroxylase (CYP7A1), which generates cholalic acid (CA) and chenodeoxycholic acid (CDCA), and an alternative pathway catalyzed by sterol 27-hydroxylase (CYP27A1), which synthesizes CDCA (Yunxia Yang, 2020). BA synthesized by these two pathways is called primary BA. Primary BA is combined with taurine and glycine to form CBA, which is secreted into the bile and further excreted into the intestine to help emulsify dietary lipids. Under the action of intestinal microbiota, primary BAs form secondary BAs, including deoxycholic acid (DCA) from CA and lithocholic acid (LCA) and ursodeoxycholic acid (UDCA) from CDCA (A. Xie et al., 2021a). The synthesis of BA is dominated by the classical pathway, forming primary BA (12α-OH BA) that is hydroxylated at the carbon 12 position, while the alternative pathway synthesizes non-12α-OH BA.

The synthesis of BA in humans and mice is very similar, but the composition of BA and the size of the pool vary substantially. As in humans, CA is formed in mice through the classical pathway. In addition to CDCA, UDCA is also synthesized through alternative pathways in mice and further hydroxylated at the 6β position to form α-muricholic acid (α-MCA) and β-muricholic acid (β-MCA) (Takahashi et al., 2016; Jianing Li, 2019). Unlike in humans, UDCA is a primary BA in mice, and the primary BA mainly binds to taurine. In addition, changes in the gut microbiome and BA metabolism affect the diversity of secondary BAs(Akira Honda, 2020).

**FIGURE 2:**
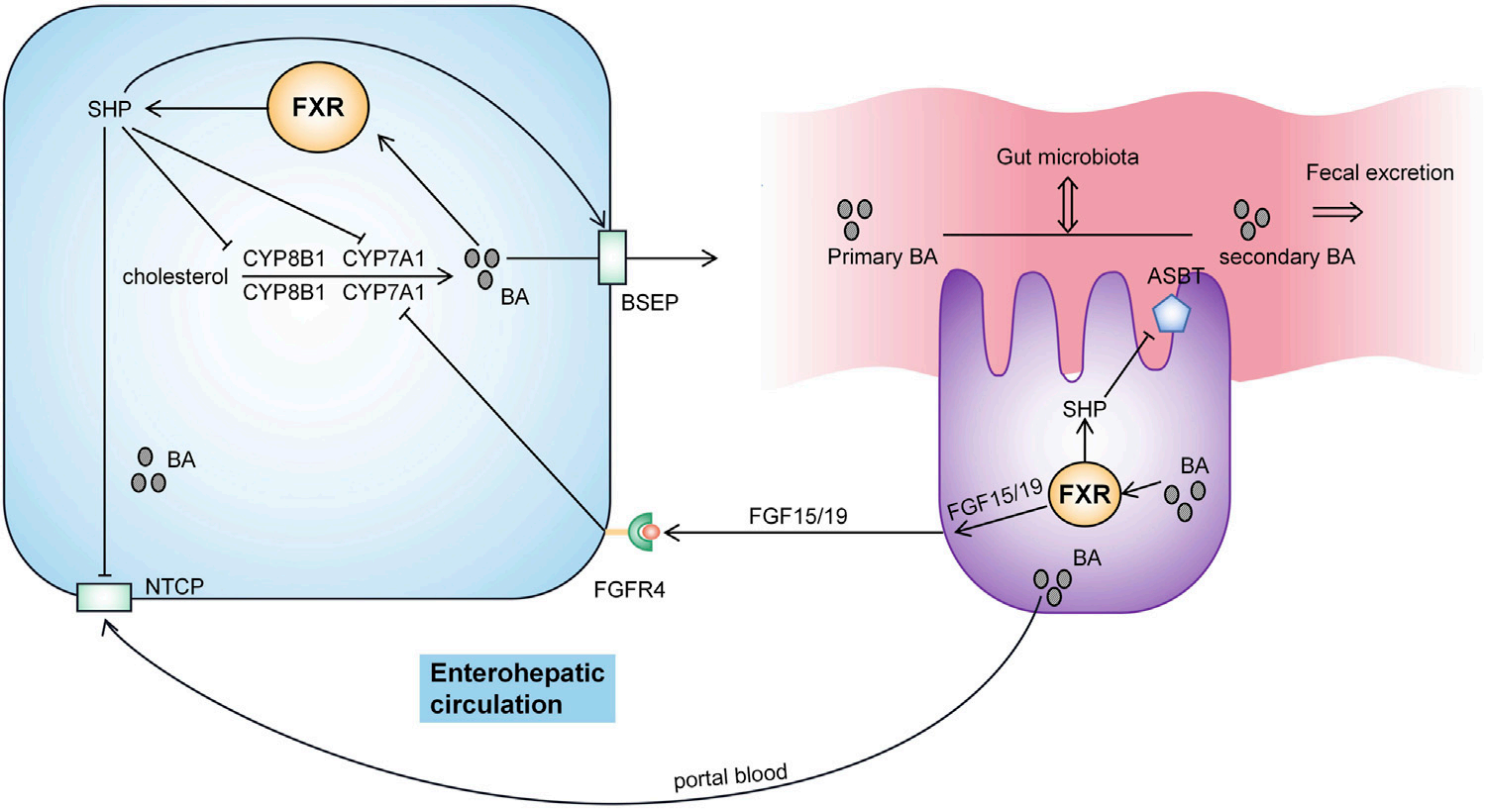
Pathway regulating BA metabolism. Hepatic synthesis of BA is a negative feedback mechanism regulated by farnesyl X receptor (FXR). BA is secreted into bile by BSEP and then excreted into the intestine. BA enters the portal vein circulation in the gut through ASBT and is then absorbed mainly by the liver cells *via* NTCP. BA activates FXR to induce small heterodimer partner (SHP) activity and inhibit the transcription of the CYP7A1 and CYP8B1 genes. Meanwhile, FXR activation increases BA excretion by upregulating the expression of BSEP and ASBT. FXR activation also inhibits NTCP *via* SHP. FGF15/19 binds to FGF receptor 4 (FGFR4) in the liver to inhibit CYP7A1 transcription and thus inhibit BA synthesis. BA interacts with the intestinal flora.

BA homeostasis is maintained through multiple negative feedback loops and the enterohepatic circulation of BA (Figure 2). Most of the BA in the intestine returns to the liver by transport in the small intestine through enterohepatic circulation (Trauner et al., 2017), and a small part (approximately 5%) is excreted through feces or reaches the systemic circulation to participate in BA signaling outside the intestinal-liver system and regulate metabolism, inflammation and the intestinal flora (Shapiro et al., 2018). Hepatic synthesis of BA is a negative feedback mechanism regulated by farnesyl X receptor (FXR) (Choudhuri and Klaassen, 2022). Activation of FXR by BA induces small heterodimer partner (SHP) to inhibit the transcription of CYP7A1 and CYP8B1 genes, reducing BA synthesis (Ding et al., 2015). CBA is secreted into bile by the bile salt export pump (BSEP) and then excreted into the intestine. BA enters the portal vein circulation in the gut through the apical sodium-dependent bile acid transporter (ASBT) and is then absorbed mainly by liver cells *via* (Na+)-taurocholate cotransporting polypeptide (NTCP) (Chiang and Ferrell, 2020). FXR activation increases BA excretion by upregulating the expression of the tubular transporter proteins BSEP and ASBT and by inhibiting NTCP through SHP (Denson et al., 2001; Ding et al., 2015). In enterocytes, FGF15/19 is an endocrine hormone secreted from the terminal ileum and its levels are regulated by both FXR and BA. FGF15/19 binds to FGF receptor 4 (FGFR4) in the liver to inhibit CYP7A1 transcription and subsequently inhibit BA synthesis (Katafuchi and Makishima, 2022). In the gut, BA interacts with the intestinal microbiota (Chen et al., 2019). The composition of the gut microbiota determines the uncoupling, dehydroxylation, and oxidation of secondary BAs and regulates BA metabolism and synthesis in an FXR-dependent manner (Sayin et al., 2013). BA regulates the intestinal microbial composition by activating innate immunity-related genes in the intestine (Wahlström et al., 2016).

## 3 The metabolic regulatory effect of BA

Conversion to BA in the liver is one of the most important methods to eliminate cholesterol in the body. Dysregulation of BA synthesis and metabolism is associated with obesity, T2DM, NAFLD and other metabolic diseases (Guan et al., 2022). BA is not only involved in the digestion and absorption of lipids and lipid-soluble nutrients in the intestine but also regulates lipid and glucose metabolism through FXR and TGR5 and inflammatory reactions in the liver and other tissues (Molinaro et al., 2018). FXR is involved in regulating lipid metabolism, particularly the transport, synthesis, and utilization of TGs. BA affects FXR-mediated signaling in the gut and liver, and higher concentrations of BA regulate lipid homeostasis by activating FXR (Katafuchi and Makishima, 2022). In addition to regulating BA metabolism, FGF15/19 also regulate glycogen synthesis and cholesterol catabolism (Kim et al., 2020). The gut microbiota has been shown to affect glucose and lipid metabolism by regulating the BA signaling pathway (Wang et al., 2022). Alternative pathways are increasingly being shown to play key roles in regulating lipids, cholesterol, carbohydrates, and energy homeostasis. Activation of alternative synthetic pathways improves metabolism (Jia et al., 2021).

## 4 The role of BA metabolism in NAFLD

### 4.1 Changes in BA levels in individuals with NAFLD/NASH

The occurrence of NAFLD is clearly associated with BA metabolism, but the specific changes have not been conclusively determined. Most studies suggest that BA levels are elevated in the blood and liver of NAFLD patients. Some studies also showed that NAFLD patients did not exhibit a difference in total bile acid (TBA) levels compared with healthy people, but the BA composition changed substantially (Caussy et al., 2019). BA metabolism is associated with insulin resistance (Haeusler et al., 2013), but dysregulated BA metabolism in individuals with NAFLD is independent of obesity and the T2DM status, with dysregulation being more prominent in non-obese NAFLD patients (Jung et al., 2021). NASH patients have significantly increased TBA and concentrations of both primary BAs and secondary BAs (Ferslew et al., 2015; Jiao et al., 2018; Kalhan et al., 2011). BA levels are substantially changed in the hepatic-intestinal circulation in NASH patients, the effects of which can be corrected with diet (Gillard et al., 2022).

The role of alternative pathway activation in regulating metabolism is constantly being recognized, and the increased ratio of 12-OH-BA/non-12-OH-BA promotes metabolic disease (Sang et al., 2021). Studies (G. Xie et al., 2021b) have shown that liver fibrosis in NASH patients is associated with significantly elevated liver levels of 12α-OH BAs, such as taurine deoxycholate (TDCA) and glucose deoxycholate (GDCA). 12α-OH BA significantly promotes liver fibrosis through TGR5-mediated p38MAPK and ERK1/2 signaling. Additionally, plasma levels of the 7-keto-BAs are dose-dependently altered and associated with NASH and balloon-like changes, and the 7-keto-DCA levels were also shown to be associated with advanced stages of liver fibrosis (Nimer et al., 2021).

Abnormal BA metabolism during the development of NAFLD is regulated by complex pathways. Changes in BA metabolizing enzymes and transporter expression that occur with the progression of NASH-related liver fibrosis in mouse NAFLD models have been shown to cause increased TBA concentrations in plasma (Suga et al., 2019). Hepatic expression of the BA transporter is downregulated due to inflammation, which slows BA enterohepatic circulation and leads to increased BA levels in the serum and liver of patients with NASH (Tanaka et al., 2012; Phillip B Hylemon, 2021). Levels of the FXR antagonist DCA are increased in individuals with NAFLD, while levels of the agonist CDCA are decreased, FXR is inhibited, and BA signaling is inhibited (Jiao et al., 2018). Instead, inhibition of BA reabsorption increases fecal BA excretion and the mRNA expression of genes related to BA synthesis in the liver, increasing *de novo* BA synthesis from cholesterol and ameliorating NAFLD induced by a high-fat diet (Rao et al., 2016).

### 4.2 Roles of the intestinal flora in the development of NAFLD and BA metabolism

NAFLD patients have abnormalities in the intestinal flora and the expression of genes related to BA metabolism. NAFLD patients exhibit a lower bacterial diversity, and the abundance of *Bacteroides* was mainly decreased in NAFLD patients (Jiao et al., 2021). Even the maternal dietary structure affects BA metabolism in offspring. When perinatal mice were fed a high-salt diet, their offspring showed greater changes in fecal microbial β-diversity, which further promoted a BA imbalance, increased intestinal barrier permeability and reduced hepatic FXR expression, thus promoting NAFLD (Guo et al., 2021). Abnormal interactions of the microbiome, BA and FXR were involved in the occurrence of NAFLD.

### 4.3 The role of BA metabolism in NAFLD-LC/HCC

Studies examining BA metabolism in NASH-LC/HCC are currently relatively limited. The results from mouse and *in vitro* studies have shown that TDCA and GDCA effectively activate hepatic stellate cells and promote LC (G. Xie et al., 2021b). A clinical study showed that serum levels of the primary CBAs G(T)CA and G(T)CDCA were significantly increased in NASH-LC patients compared with NASH patients without LC, which was independent of the occurrence of HCC (Sydor et al., 2020).

The metabolic disorders induced by HCC are mainly related to primary BA biosynthesis. Abnormal accumulation of BA distorts macrophage polarization and generates an immunosuppressive tumor microenvironment (Sun et al., 2022). Few studies have assessed the role of BA in HCC after NAFLD. A potential transition in the alternative BA synthesis pathway mediated by increased CYP7B1 mRNA and protein expression was observed in subjects with NASH(Lake et al., 2013), which is presumed to be a self-protective mechanism in the liver, but its products may play a role in NASH-HCC(Jia et al., 2021). Cholesterol transport to mitochondria by steroidogenic acute regulatory protein 1 (STARD1) is an essential component of an alternative pathway for BA production. STARD1 stimulates BA production through the mitochondrial alternative pathway, and its products also play a key role in NASH-HCC(Conde De La Rosa et al., 2021). UDCA and obeticholic acid ameliorate HCC(Luo et al., 2022), providing new ideas for the treatment of HCC.

## 5 Analysis of NAFLD treatments related to BA metabolism

The treatment of NAFLD is still mainly based on lifestyle adjustments, and an effective drug is not available. Drugs that regulate BA metabolism have been used in the clinic to ameliorate hyperlipidemia (Lee et al., 2022). Recently, approaches interfering with BA synthesis and metabolism have become a new research direction for the treatment of NAFLD (Trauner and Fuchs, 2021) (Table 1). Inhibition of ileal BA uptake promotes BA synthesis, reduce hepatic triglyceride (TG) and cholesterol contents, and prevents NAFLD in mice fed a high-fat diet (Rao et al., 2016). Animal experiments have shown that CYP8B1 knockdown leads to resistance to weight gain and hepatic steatosis caused by a Western diet (Bertaggia et al., 2017).

**TABLE 1:** Drugs and their effects on NAFLD treatment by regulating BA.

Drug category	Medicines	Function
FXR agonist	GSK2324, obeticholic acid	Reduced BA synthesis
BAS	Colesevelam, cholestyramine	Increased BA excretion
BA transporter protein inhibitors	Elobixibat	Lowering serum BA levels, increasing fecal BA concentrations and improving microflora dysregulation
Gut microflora and its modulators	Flaxseed powder	Activation of the intestinal FXR-FGF15 and TGR5-NF-κB pathways and regulation of BA metabolism
	Biambiguous triple capsules	Effects on BA metabolism
	*Akkermansia muciniphila* and quercetin	Promotion of the hepatic synthesis and transport of BA
	*L. rhamnosus* LGG	Inhibition of *de novo* BA synthesis and increased BA excretion
TCM and traditional Chinese medicine ingredients	Glycyrrhizin	Restoration of BA homeostasis
		Inhibition of inflammatory injury
	Hyperin	Regulation of BA metabolism and excretion
	Dihydroflavonoids	Regulation of BA metabolism

FXR agonists can reduce lipotoxicity by promoting mitochondrial β oxidation, reducing *de novo* adipogenesis, and stimulating cholesterol excretion. Application of the FXR agonist GSK2324 prevents NAFLD by selectively reducing BA synthesis and reducing lipid absorption (Clifford et al., 2021). Obeticholic acid, an FXR activator, has been shown to improve hepatic steatosis and reduce liver inflammation and fibrosis (Younossi et al., 2019). BA sequestrant (BAS), which binds BA in the intestine and promotes its fecal excretion, has long been used in the clinic to reduce LDL cholesterol levels and has now been shown in animal experiments to reverse liver damage and prevent NAFLD progression in mice fed a Western diet (Takahashi et al., 2020). In addition, traditional Chinese medicine (TCM) and TCM components have also been shown to attenuate NAFLD by regulating BA metabolism. Hypericin, a common ingredient of TCM with antioxidant-protective, hepatoprotective and anti-inflammatory effects, has been shown to improve the status of NAFLD by regulating cholesterol and BA metabolism (Wang et al., 2021). Glycyrrhizin has been shown to regulate BA metabolism and subsequently improve NASH by restoring hepatic FXR inhibition (Yan et al., 2018). As a bioactive component of a traditional Chinese herbal medicine, dihydroflavonoids were shown to significantly reduce CYP7A1 expression, suggesting that they ameliorate NAFLD by regulating BA metabolism (Li et al., 2022).

Interfering with the composition of intestinal microorganisms to regulate the composition of BA, liver metabolism and inflammation has also become a new direction for the treatment of NAFLD. Supplementation with *Akkermansia muciniphila* and quercetin regulates the intestinal microbiota of mice, promotes the liver synthesis and transport of BA, and attenuates NAFLD (Juárez-Fernández et al., 2021). The consumption of biambiguous triple capsules has been shown to affect BA metabolism and reduce liver enzyme levels (Zhou et al., 2021). The consumption of flaxseed powder has been shown to regulate the intestinal microbiota and activate intestinal FXR-FGF15 and TGR5-NF-κB pathways to regulate BA metabolism and improve NASH(Yang et al., 2021). An ileal bile acid transporter inhibitor (IBATi) reduces serum BA levels and increase fecal BA concentrations. IBATi reduces proinflammatory factor expression in the liver and attenuates hepatic steatosis, inflammation and fibrosis by improving intestinal dysbiosis in NAFLD model mice (Matsui et al., 2021; Yamauchi et al., 2021). An increasing number of drugs that interfere with BA metabolism and BA-related signaling pathways have been suggested to treat metabolism-related diseases, and more studies are needed to clarify their effects and safety in the future.

## 6 Prospects and summary

In conclusion, NAFLD imposes a huge medical burden worldwide. BA plays an important role in regulating metabolism. The role of abnormal BA metabolism in the occurrence and development of NAFLD and its associated LC/HCC cannot be ignored. BA levels are increased in the blood of NAFLD/NASH patients, generally including both CBA and FBA, but different research results have also been reported. Few studies have reported changes in BA metabolism in the liver of NAFLD/NASH patients, and the results are not completely consistent. Therefore, changes in BA metabolism during the development of NAFLD/NASH and its role in the disease still remain to be explored.

A large number of patients are diagnosed with NAFLD, and the identification of patients who can easily develop LC or HCC is very important. BA has good utility in assessing NAFLD progression in patients with T2DM (Wu et al., 2021), but BA is rarely studied in diagnostic models of NAFLD. BA profiles show significant differences in patients with chronic liver diseases with different causes (Sang et al., 2021), suggesting the clinical potential of BA profiles in the differentiation of liver injury types. Retrospective studies have also shown that primary BA is associated with future liver-related events in individuals with NAFLD (Wegermann et al., 2021), suggesting the potential of BA metabolism for predicting the NAFLD prognosis. Further studies are needed to determine whether the future NAFLD severity can be assessed by detecting BA metabolomic indicators. BA metabolism changes in patients with NAFLD, and correcting this change may improve NAFLD. Further studies are needed to assess whether NAFLD can be treated by intervening in BA metabolism in clinical practice.
